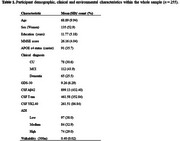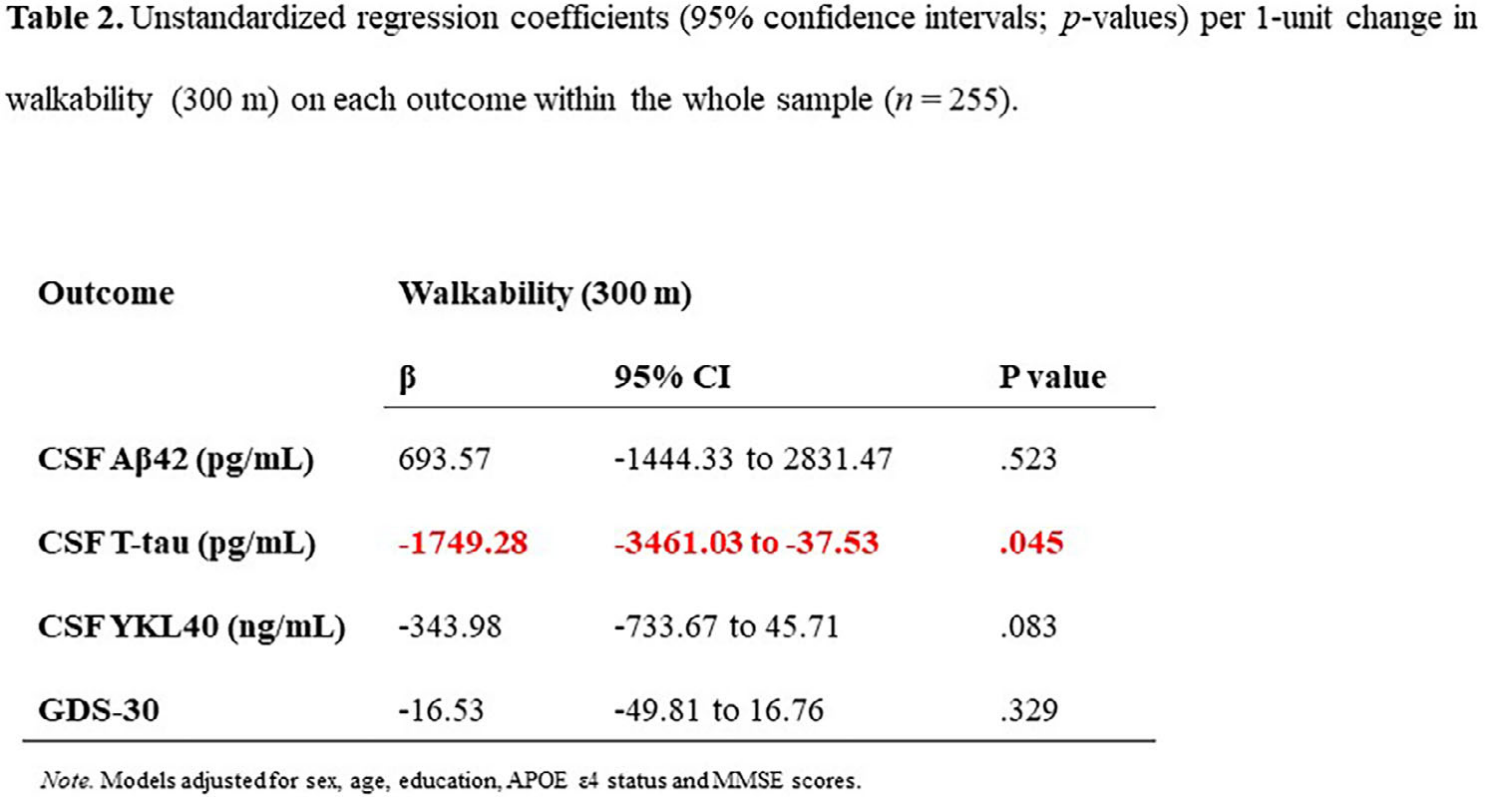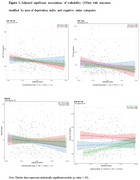# Association of neighbourhood walkability with pathophysiological markers and depressive symptoms by area deprivation index and cognitive status in older adults

**DOI:** 10.1002/alz70860_105259

**Published:** 2025-12-23

**Authors:** Eleni Palpatzis, Pablo Aguilar, Carmen M Colceriu, Alex López, Sara E Zsadanyi, María Franquesa‐Mullerat, Sami Petricola, Antonia Valentín, Alberto Lleó, Juan Fortea, Alexandre Bejanin, Eider M Arenaza‐Urquijo

**Affiliations:** ^1^ Global Health Institute Barcelona (ISGlobal), Barcelona, Spain; ^2^ Universitat Pompeu Fabra, Barcelona, Spain; ^3^ ISGlobal, Barcelona, ‐, Spain; ^4^ University of Pompeu Fabra (UPF), Barcelona, Spain; ^5^ Sant Pau Memory Unit, Department of Neurology, Hospital de la Santa Creu i Sant Pau, Institut d'Investigació Biomèdica Sant Pau (IIB SANT PAU), Facultad de Medicina ‐ Universitat Autònoma de Barcelona, Barcelona, Spain; ^6^ Center of Biomedical Investigation Network for Neurodegenerative Diseases (CIBERNED), Madrid, Spain; ^7^ CIBERNED, Network Center for Biomedical Research in Neurodegenerative Diseases, National Institute of Health Carlos III, Madrid, Spain; ^8^ Sant Pau Memory Unit, Hospital de la Santa Creu i Sant Pau, Institut de Recerca Sant Pau ‐ Universitat Autònoma de Barcelona, Barcelona, Spain; ^9^ Center for Biomedical Investigation Network for Neurodegenerative Diseases (CIBERNED), Madrid, Madrid, Spain; ^10^ Catalan Foundation for Down Syndrome, Barcelona, Spain; ^11^ ISGlobal ‐ Barcelona Institute for Global Health, Barcelona, Catalunya/Barcelona, Spain

## Abstract

**Background:**

Previous evidence suggests that urban design features, like neighbourhood walkability, are associated with cognitive outcomes and mental well‐being in older adults. Here, we examined associations between walkability and cerebrospinal fluid (CSF) markers of amyloid, neurodegeneration, and neuroinflammation, as well as depressive symptoms, considering neighbourhood deprivation and cognitive status.

**Method:**

We analysed cross‐sectional data from 255 participants (both cognitively impaired and unimpaired) from the Sant Pau Initiative on Neurodegeneration (SPIN) cohort. The predictors included walkability assessed within 300m radius around participants’ residences. Outcomes included CSF biomarkers of AD pathology (Aβ42), neurodegeneration (T‐tau), and neuroinflammation (YKL‐40), as well as depressive symptoms (Geriatric Depression Scale; GDS‐30). Participants also had data on neighbourhood deprivation levels measured with the Area Deprivation Index (ADI). We performed multiple linear regression models to examine associations between walkability and the outcomes, with stratification by neighbourhood deprivation level (low, medium, high) and cognitive status (CU, MCI and Dementia). All models were adjusted for age, sex, education, APOE ε4 status, and MMSE scores.

**Result:**

Participants’ age ranged from 45 to 87, 53% were women, and 26% were diagnosed with dementia (39% AD, 18% Lewy Body and 17% Frontotemporal Dementia; Table 1). In the whole sample, higher walkability was only associated with lower T‐tau levels, with no associations observed for other outcomes (Table 2). Stratified analyses by area deprivation, showed that higher walkability was associated with lower T‐tau and YKL‐40 levels among individuals living in areas with higher ADI. Additionally, higher walkability was marginally associated with lower GDS‐30 scores among individuals in medium‐deprivation areas. Stratified analyses by cognitive status, showed that higher walkability was associated with lower Aβ burden only among individuals with dementia (Figure 1).

**Conclusion:**

Our findings link neighbourhood walkability to neurodegeneration, as well as neuroinflammation and depressive symptoms in highly and moderately deprived areas and to lower Aβ in individuals with a dementia diagnosis. Future research should explore the mediating mechanisms linking walkability to brain and mental health, including physical activity and mental health promotion. Urban planning strategies may consider both walkability and neighbourhood socioeconomic context to optimize cognitive and mental health outcomes in aging populations.